# A Modified Fibronectin Type III Domain-Conjugated, Long-Acting Pan-Coronavirus Fusion Inhibitor with Extended Half-Life

**DOI:** 10.3390/v14040655

**Published:** 2022-03-22

**Authors:** Qianyu Duan, Shuai Xia, Fanke Jiao, Qian Wang, Rui Wang, Lu Lu, Shibo Jiang, Wei Xu

**Affiliations:** 1 Key Laboratory of Medical Molecular Virology (MOE/NHC/CAMS), School of Basic Medical Sciences, Shanghai Frontiers Science Center of Pathogenic Microbes and Infection, Shanghai Institute of Infectious Disease and Biosecurity, Fudan University, Shanghai 200032, China; 19111010059@fudan.edu.cn (Q.D.); sxia15@fudan.edu.cn (S.X.); 20111010053@fudan.edu.cn (F.J.); wang_qian@fudan.edu.cn (Q.W.); 2 Beijing Prosperous Biopharm Company, Beijing 100021, China; wangr@prospbiopharm.cn

**Keywords:** SARS-CoV-2, human coronavirus, fusion inhibitor, albumin, long-acting

## Abstract

The coronavirus disease 2019 (COVID-19) pandemic caused by infection of SARS-CoV-2 and its variants has posed serious threats to global public health, thus calling for the development of potent and broad-spectrum antivirals. We previously designed and developed a peptide-based pan-coronavirus (CoV) fusion inhibitor, EK1, which is effective against all human CoVs (HCoV) tested by targeting the HCoV S protein HR1 domain. However, its relatively short half-life may limit its clinical use. Therefore, we designed, constructed, and expressed a recombinant protein, FL-EK1, which consists of a modified fibronectin type III domain (FN3) with albumin-binding capacity, a flexible linker, and EK1. As with EK1, we found that FL-EK1 could also effectively inhibit infection of SARS-CoV-2 and its variants, as well as HCoV-OC43. Furthermore, it protected mice from infection by the SARS-CoV-2 Delta variant and HCoV-OC43. Importantly, the half-life of FL-EK1 (30 h) is about 15.7-fold longer than that of EK1 (1.8 h). These results suggest that FL-EK1 is a promising candidate for the development of a pan-CoV fusion inhibitor-based long-acting antiviral drug for preventing and treating infection by current and future SARS-CoV-2 variants, as well as other HCoVs.

## 1. Introduction

Coronaviruses can be classified into four genera, namely α-, β-, γ-, and δ-coronavirus [1]. β-coronaviruses can be further divided into four lineages: A, B, C, and D. The seven coronaviruses that can infect humans (HCoVs) include HCoV-229E and HCoV-NL63 in the α-coronavirus family, HCoV-OC43 and HCoV-HKU1 in the β-coronavirus lineage A, SARS-CoV and SARS-CoV-2 in the β-coronavirus lineage B, and MERS-CoV in the β-coronavirus lineage C [2]. Coronavirus disease 2019 (COVID-19) caused by SARS-CoV-2 infection has spread worldwide since December 2019. Recently, a series of SARS-CoV-2 variants of concern (VOC), including Alpha (B.1.1.7) [3], Beta (B.1.351) [4], Gamma (P.1), Delta (B.1.617.2) [5], and Omicron (B.1.1.529) [6] have emerged with enhanced transmissibility and/or decreased sensitivity to the current COVID-19 vaccines and antibody therapeutics [7,8]. This calls for the development of broad-spectrum anti-HCoV agents [9].

We previously discovered a pan-coronavirus (pan-CoV) fusion inhibitor, EK1, which could potently inhibit the infection of SARS-CoV, MERS-CoV, SARS-CoV-2, and other HCoVs by targeting the HR1 domain in the S2 subunit of the S protein [10]. However, its future clinical application is limited by its short half-life in blood circulation. This is the result of kidney filtration, whereby molecules with molecular weights less than 40–50 kDa are rapidly removed from the blood [11]. Therefore, extending the circulating plasma half-life of EK1 would improve its pharmacokinetic property and, thus, lead to the development of long-acting antivirals to prevent and treat current infection by SARS-CoV-2 and its variants, as well as emerging HCoV infections [12].

One of the widely used applications to extend the half-life of a peptide or protein drug is to conjugate the polyethylene glycol (PEG) to the N- or C-terminus of the peptide or protein, in order to reduce the speed of spherical filtration and elimination of the peptide, thus increasing its plasma half-life [13]. However, the use of a PEG linker in a protein or peptide drug may cause anti-PEG antibodies in vivo that can reduce the therapeutic efficacy of the drug or increase its adverse risks [14]. Instead of the PEG strategy, we prefer to conjugate the small molecule monomer fibronectin type III structural domain (FN3) to a peptide or protein for extending the half-life of the peptide or protein because (1) FN3 has a small molecular weight, easy mass expression, stable physicochemical properties, and high biosafety, (2) the variable surface region of FN3 possesses a targeted-binding capacity similar to that of an antibody, and (3) the FN3-conjugated peptide or protein can reversibly bind to the serum albumin, resulting in the extension of its half-life [15,16,17,18]. 

Therefore, we have designed, constructed, and expressed a recombinant protein designated FL-EK1, which comprises a modified fibronectin type III structural domain (FN3) with albumin-binding capacity, a linker, and EK1. We found that FL-EK1 expressed in *E. coli* was soluble and could reversibly bind to human serum albumin (HSA) with high binding affinity. We demonstrated that FL-EK1, like EK1, could effectively inhibit SARS-CoV-2 spike protein-mediated cell–cell fusion and suppress infection by both pseudotyped and authentic SARS-CoV-2 and its Delta variant, as well as HCoV-OC43. As with EK1, FL-EK1 could also protect mice from infection by authentic SARS-CoV-2 and HCoV-OC43. By measuring ex vivo inhibitory activity, we found that the half-life of FL-EK1 in blood circulation was about 15.7-fold longer than that of EK1, suggesting that FL-EK1 has the potential to be developed as a long-acting pan-CoV fusion inhibitory prophylactic or therapeutic against infection by current and future SARS-CoV variants and other HCoVs.

## 2. Materials and Methods

### 2.1. Cell Lines, Viruses, Peptides

293T, Caco2, Calu3, and RD cells were obtained from the American Type Culture Collection (ATCC). Huh-7 cells were obtained from the Cell Bank of the Chinese Academy of Sciences (Shanghai, China). All cell lines were maintained and grown in Dulbecco’s Modified Eagle’s Medium (DMEM, Invitrogen, Waltham, MA, USA) containing 100 U/mL penicillin, 100 mg/mL streptomycin, and 10% fetal bovine serum (FBS). 

Authentic SARS-CoV-2 wild type (nCoV-SH01) and SARS-CoV-2 variant Delta (B.1.617.2) were isolated from patients in Shanghai and maintained at the Biosafety Level 3 (BSL-3) Laboratory of Shanghai Medical College, Fudan University [19]. 

**Figure 1:**
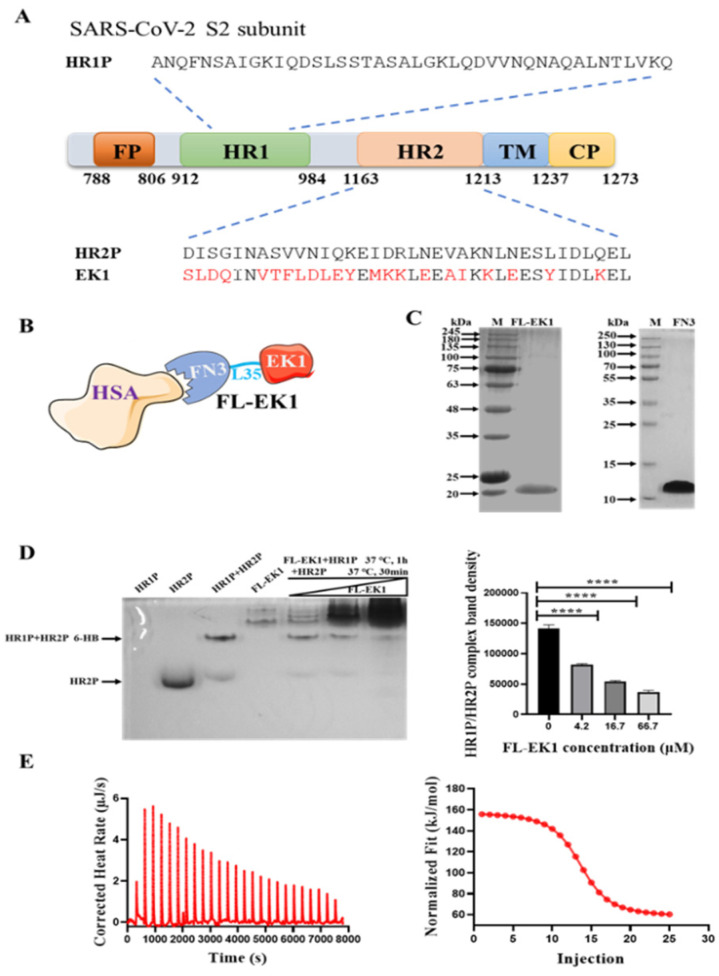
Design, construction, and characterization of FL-EK1. (**A**) Schematic diagram of SARS-CoV-2 S2 subunit and sequences of the peptides HR1P, HR2P, and EK1. The amino acids highlighted in red in EK1 represent amino acids that are different from those in HR2P derived from the SARS-CoV-2 S2 subunit. FP, fusion peptide; HR, heptad repeat; TM, transmembrane region; CP, cytoplasm region. (**B**) Diagram of FN3-conjugated EK1 (FL-EK1) binding to HSA. (**C**) SDS-PAGE analysis of the purified FL-EK1 and FN3 proteins. (**D**) FL-EK1 inhibition of 6-HB formation between HR1P and HR2P, as determined by N-PAGE. **** *p* < 0.0001. (**E**) Binding affinity of FL-EK1 to HSA, as evaluated by isothermal titration calorimetry (ITC) assay. Data were analyzed and processed by TA-ITC software.

SARS-CoV-2 HR1 peptide, HR2 peptide, and EK1 peptide [10] were synthesized by staff at the Chengdu Shengnuo Biotechnology Co., Ltd. (Chengdu, China). Their sequences are shown in Figure 1A. 

### 2.2. Plasmids

The luciferase reporter vector (pNL4-3.Luc.R-E-), the envelope-expressing plasmids of SARS-CoV-2-S (pcDNA3.1-SARS-2-S, GenBank: MT079854.1), HCoV-OC43-S (pcDNA3.1-OC43-S, GenBank: CAA83661.1), the envelope-expressing plasmids of SARS-CoV-2 variant-S (pcDNA3.1-P.1-S, GenBank: MZ477859.1; pcDNA3.1-B.1.1.7-S, GenBank: OM616632.1; pcDNA3.1-B.1.525-S, GenBank: MZ362451.1; pcDNA3.1-B.1.617.2-S, GenBank: OK091006.1; pcDNA3.1-B.1.351-S, GenBank: MZ433432.1; pcDNA3.1-B.1.617.1-S, GenBank: MZ571142.1; pcDNA3.1-C.37-S, GenBank: MZ275302.1; and pcDNA3.1-B.1.1.529-S, GenBank: OM570283.1), as well as pAAV-IRES-EGFP plasmids that encode EGFP, were maintained in our laboratory [20].

### 2.3. Expression and Purification of FL-EK1

The FL-EK1 gene was synthesized and subcloned into pET-28a (+) vector (Prosperous Biopharm Co., Ltd., Beijing, China) for expression in *E. coli* strain BL21 (DE3) (Thermo Scientific, Carlsbad, CA, USA). The engineered bacteria were cultured in LB medium containing 100 μg/mL of kanamycin at 30 °C to an optical density (OD_600_) of 0.6 induced with 0.2 mM isopropyl-β-d-thiogalactopyranoside (IPTG) at 16 °C for 12 h. Then, the harvested bacteria were resuspended in a binding buffer containing 10 mM imidazole and subsequently lysed by ultrasonication on ice. The lysate was centrifuged and purified by Ni-NTA (Qiagen, GmbH, Hilden, Germany). The purified protein was dialyzed against PBS to remove imidazole and was examined by Coomassie blue staining separated on an SDS–PAGE gel.

### 2.4. Native Polyacrylamide Gel Electrophoresis (N-PAGE) Analysis of FL-EK1

N-PAGE was performed to detect 6-HB formation between the HR1P (120 μM) and HR2P (40 μM) peptides of SARS-CoV-2 and assess the effect of FL-EK1 at 4.2, 16.7, and 66.7 μM on the formation of 6-HB between the HR1P and HR2P peptides. Serially diluted FL-EK1 (4.2, 16.7, and 66.7 μM) was incubated with an equal volume of SARS-CoV-2 HR1P (120 μM) at 37 °C for 1 h. HR2P (40 μM) was then added and incubated at 37 °C for 0.5 h. Each mixture was loaded onto a Tris-glycine gel (18%). After running the gel for 3 h, it was visualized by Coomassie blue.

### 2.5. Isothermal Titration Calorimetry (ITC) Analysis of FL-EK1 Binding to HSA

An ITC assay was used to detect the interaction between FL-EK1 and HSA with an ITC microcalorimeter instrument (NANO ITC, TA Instruments, Newcastle, DE, USA). In brief, HSA was dissolved in PBS to 350 μM and injected into the chamber containing 35 μM of FL-EK1. Titration was performed at a constant temperature of 25 °C and a stirring rate of 220 rpm. Twenty-five injections were automatically performed with an injection interval of 300 sec. After that, TA-ITC software was used to analyze the data.

### 2.6. Assay for Assessing the Inhibition of Pseudotyped HCoV Infection

Pseudoviral experiments were carried out to assess whether the FL-EK1 protein has broad-spectrum anti-HCoV activity. The pNL4-3.Luc.R-E- (luciferase reporter-expressing HIV-1 backbone) and pcDNA3.1 plasmids, separately encoding SARS-2-S, HCoV-OC43-S, P.1-S, B.1.1.7-S, B.1.525-S, B.1.617.2-S, B.1.351-S, B.1.617.1-S, C.37-S, and B.1.1.529-S, were co-transfected with 293T cells using VigoFect (Vigorous Biotechnology, Beijing, China). The supernatant containing the pseudotyped coronavirus was harvested and centrifuged at 3000 rpm for 10 min and filtered with a 0.45 μm sterilizing filter, followed by storage at −80 °C. The pseudotyped HCoV infection experiment was performed as described previously [10]. Briefly, target cells (RD cells for HCoV-OC43; Calu3/Caco2 cells for SARS-CoV-2 and variants) were seeded in a 96-well plate (1 × 10^4^ cells/well). After 12 h, a pseudotyped HCoV was mixed with an equal volume of FL-EK1, which was serially diluted in DMEM medium and incubated at 37 °C for 30 min. The supernatant of the cell culture was removed, and the inhibitor/virus mixture (100 μL) was added to the target cells. The supernatant was changed after 12 h and then incubated for an additional 48 h. Cells were lysed with a lysis reagent (Promega, Madison, WI, USA), followed by luminescence detection with a Multi-Detection Microplate Reader using the Luciferase Assay System (Promega, Madison, WI, USA).

### 2.7. Assay for Evaluating S Protein-Mediated Inhibition of Cell–Cell Fusion

S protein-mediated cell–cell fusion was performed as previously described [10]. In brief, 293T cells were transfected with pAAV-IRES-EGFP vector plasmid encoding SARS-CoV-2 S or HCoV-OC43 S proteins as the effector cells. About 12 h later, 293T/S/GFP cells were mixed with equal volume of 3-fold serially diluted FL-EK1, EK1, and FN3 in a round-bottom 96-well plate and incubated at 37 °C for 30 min. The mixture was added to Huh-7/RD cells as the target cells (2 × 10^4^ cells/well) at 37 °C for 2~6 h. The fused cells were observed and counted using the green fluorescence channel of a fluorescence microscope, and the IC_50_ value was calculated.

### 2.8. Assay for Testing the Inhibition of Authentic HCoV Infection

The experiment for assessing the inhibition of authentic SARS-CoV-2 and Delta variant was carried out in a BSL-3 Laboratory in Shanghai Medical College of Fudan University. The inhibitory activity of FL-EK1 on SARS-CoV-2 was determined by qPCR. FL-EK1 diluted in culture medium at an indicated concentration was added to a round bottom 96-well plate, and an equal volume of authentic SARS-CoV-2 WT strain or Delta variant at 100 TCID_50_ was added. The plate was incubated at 37 °C for 30 min, and then the mixture was transferred into the wells of pre-cultured Caco2 cells, the original medium of which was discarded. After 12 h of incubation at 37 °C, the culture medium was replaced with 2% FBS DMEM, and the culture was continued for an additional 48 h. The culture supernatant was collected and put in prepared EP tubes containing TRIzol™ LS Reagent (InvitrogenTM, Waltham, MA, USA), followed by storage at −80 °C. The EasyPure Viral DNA/RNA Kit (TransGen Biotech Co., Ltd., Beijing, China) was used to extract RNA from the supernatant. Reverse-transcription quantitative PCR (RT-qPCR) was used to test SARS-CoV-2 or Delta RNA copies by using the One Step PrimeScript RT-PCR Kit (Takara, Shiga, Japan). The primers and probe for detection of N gene genomic mRNA were as follows: SARS-CoV-2 N-F: 5′-GGGGAACTTCTCCTGCTAGAAT-3′; SARS-CoV-2 N-R: 5′-CAGACATTTTGCTCTCAAGCTG-3′; SARS-CoV-2 N-probe: 5′-FAM-TTGCTGCTGCTTGACAGATT-TAMRA-3′. 

The Cell Counting Kit-8 (CCK8, Dojindo, Japan) assay was used to evaluate whether FL-EK1 has anti-HCoV-OC43 activity. Briefly, after FL-EK1-treated cells were cultured with authentic HCoV-OC43 for a total of 60 h, as described above, CCK8 reagent was added to the cultured cells and incubated at 37 °C for 4 h before A450 was measured with the Multi-Detection Microplate Reader (Tecan, Maennedorf, Switzerland) and before the IC_50_ was calculated to measure the inhibition of FL-EK1 on cytopathic effect caused by HCoV-OC43 infection.

### 2.9. Assays to Evaluate the In Vivo Efficacy of FL-EK1 against HCoV-OC43 and SARS-CoV-2 in Mouse Infection Models

ICR newborn mice to be used in the protective study of FL-EK1 against authentic HCoV-OC43 infection were bred from pregnant mice purchased from the Beijing Vital River Laboratory Animal Technology Co., Ltd. (Beijing, China). All related experiments were carried out in strict accordance with institutional regulations. Each group had 11 3-day-old newborn mice. To evaluate the prophylactic and therapeutic effect of FL-EK1 on HCoV-OC43-infected mice, FL-EK1 (10 mg/kg) was administered intranasally 0.5 h before challenge (for prevention) or 0.5 h after challenge (for treatment) via the intranasal route with authentic HCoV-OC43 at a dose of 100 TCID_50_. For the non-treatment control group, the same volume of PBS was administered intranasally. On the fifth day after infection, the brain tissues of mice were collected and stored. The viral titer in mice brains was assessed as described [21]. The primers for detection of genomic mRNA were as follows: HCoV-OC43 S-F: 5′-GACACCGGTCCTCCTCCTAT-3′; HCoV-OC43 S-R: 5′-ACACTTCCCTTCAGTGCCAT-3′; HCoV-OC43 GAPDH-F: 5′-TGCTGTCCCTGTATGCCTCTG-3′; HCoV-OC43 GAPDH-R: 5′-TTGATGTCACGCACGATTTCC-3′.

Eight-week-old SPF Tgtn (CAG-humanACE2-IRES-Luciferase) mice purchased from Shanghai Model Organisms Center, Inc. (Shanghai, China) were used in the protective study of FL-EK1 against authentic SARS-CoV-2 Delta variant infection. The relevant experiment was carried out in the BSL-3 Laboratory of Shanghai Medical College of Fudan University in strict accordance with institutional regulations. In order to monitor the prevention and treatment effects of FL-EK1 on hACE2 transgenic mice (6 mice/group), FL-EK1 (10 mg/kg) and authentic SARS-CoV-2 Delta strain were administered intranasally in a manner similar to that with ICR mice. On the fourth day after infection, the lung, intestine, and brain tissues of the mice were collected, and RNAiso Plus (Takara) and steel balls (2 mm) were added. The tissues were then ground, and the tissue RNA was extracted. RT-PCR was performed with the One Step PrimeScript RT-PCR Kit (Perfect Real Time, Takara). The primers and probe for detection of ORF1ab gene genomic mRNA were as follows: SARS-CoV-2 ORF1ab-F: 5′-CCCTGTGGGTTTTACACTTAA-3′; SARS-CoV-2 ORF1ab-R: 5′-ACGATTGTGCATCAGCTGA-3′; SARS-CoV-2 ORF1ab-probe: 5′-FAM-CCGTCTGCGGTATGTGGAAAGGTTATGG -BHQ1-3′.

### 2.10. Study of the Half-Life of FL-EK1 and EK1

BALB/c mice (8 weeks old) were purchased from the Beijing Vital River Laboratory Animal Technology Co., Ltd. Briefly, 100 μL of FL-EK1 and EK1 (380 μM) were intraperitoneally injected into three female BALB/c mice. Then serum samples were collected before (0 h) and after injection of EK1 (0.5 h, 1 h, 3 h, 7 h, and 12 h) or FL-EK1 (0.5 h, 1 h, 3 h, 7 h, 24 h, 48 h, 72 h, and 96 h). After being kept at room temperature for 1 h, whole blood samples were centrifuged at 6000 rpm for 10 min, and the serum at the upper layer was collected and stored at −80 °C until use. Anti-SARS-CoV-2 PsV activity of mouse serum samples was determined in the same way as that described above. The highest dilution-fold of the serum causing 50% inhibition of pseudotyped SARS-CoV-2 infection (IC_50_) was calculated. To determine the inhibition of pseudotyped SARS-CoV-2 infection in mouse serum treated with FL-EK1 or EK1, the IC_50_ value of FL-EK1 or EK1 was calculated, as described above. Then, the serum half-life and other pharmacokinetic parameters of FL-EK1 or EK1 were calculated by using MODFIT software [22]. For using the MODFIT software, we selected the NCA table to enter time and concentration data for curve fitting, in order to obtain the corresponding t_1/2_ value and other pharmacokinetic parameters.

### 2.11. In Vitro Cytotoxicity and In Vivo Safety Tests

The cytotoxicity of FL-EK1 to Huh7, Caco2, RD, and Calu3 cells was measured by using the CCK8 kit, as previously described [20]. In brief, FL-EK1 at graded concentration was incubated with target cells (1 × 10^4^ cells/well) at 37 °C for 2 days before adding 100 μL of CCK8 reagent. After incubation at 37 °C for 2 h, A450 was measured with the Multi-Detection Microplate Reader, and cell viability was calculated.

To assess the in vivo safety of FL-EK1, BALB/c mice (8 weeks old) randomly assigned to 2 groups were injected intraperitoneally with FL-EK1 at 30 mg/kg (*n* = 3) or PBS (*n* = 3) as control. The bodyweight of the mice was monitored continuously for 14 days. Creatinine and ALT in the serum were measured by using the creatinine and ALT assay kits (NJJCBIO, Nanjing, China) before injection (0 h) and 1-, 3-, and 5-days post-injection, respectively. 

### 2.12. Statistical Analysis

Statistical analysis was conducted by GraphPad Prism 7.0 software. Statistical significance of obtained data between different groups was established using One-way ANOVA. Asterisks were used to indicate the statistical significance of differences between groups: * *p* < 0.05, ** *p* < 0.01, *** *p* < 0.001, **** *p* < 0.0001.

## 3. Results

### 3.1. Design, Construction, and Characterization of FL-EK1

We have previously designed and developed a pan-CoV fusion inhibitor, EK1, which targets the HR1 domain in the HCoV S protein (Figure 1A). To generate a long-acting pan-CoV fusion inhibitor, termed FL-EK1, we linked a modified FN3, as an HSA-binding motif, to EK1 through a 35-mer flexible linker, L35 (Figure 1B). The pET-28a (+) vector was used to construct FL-EK1 and its expression in *E. coli*. Results from the SDS-PAGE analysis indicated that the expressed and purified FL-EK1 and FN3 showed a single band of about 21 kDa and 12 kDa in SDS-PAGE, respectively (Figure 1C), in agreement with their calculated theoretical molecular weights. 

To clarify whether FL-EK1 shares a mechanism of action in common with EK1, we performed N-PAGE by loading HR1P (lane 1), HR2P (lane 2), HR1P/HR2P mixture (lane 3), FL-EK1 at 4.2 μM (lane 4), and the mixtures of HR1P/HR2P + FL-EK1 at 4.2 μM (lane 5), 16.7 μM (lane 6), and 66.7 μM (lane 7) to the gel, respectively, before electrophoresis. Because HR1P has a net positive charge, it migrated upward and off the gel (lane 1) (Figure 1D). However, HR2P carries a net negative charge; therefore, it migrated downward and displayed a band in the lower part of the gel (lane 2). The HR1P/HR2P mixture formed a complex that showed a strong band and a weak band corresponding to the 6-HB and HR2P in the upper band position of the gel (lane 3), suggesting that most HR2P peptide is in the 6-HB complex, while only a little HR2P peptide is in the free state. FL-EK1 showed a major band and a few minor bands in the top position of the gel (lane 4). By increasing the concentration of FL-EK1 in the HR1P/HR2P/FL-EK1 mixtures, the density of the 6-HB band was gradually decreased (lanes 5~7 in Figure 1D, left panel, and the bars in Figure 1D, right panel), suggesting that FL-EK1 can interact with HR1P to block 6-HB formation between HR1P and HR2P peptides. 

Previous studies have shown that FN3 can be modified into an artificial albumin-binding protein [23,24]. Since the binding of FN3 to serum albumin is a reversible binding process, we used isothermal titration calorimetry (ITC) to study the interaction of FL-EK1 with HSA, thus directly measuring the heat released or absorbed during the binding process. HSA (350 μM) was titrated with FL-EK1 (35 μM) at 25 °C. By measuring heat transfer during the binding process, the affinity (K_a_), binding constant (K_d_), reaction binding ratio (n), enthalpy (ΔH), and entropy (ΔS) were 1.017E6 M-1, 9.831E-7 M, 1.423, 100 KJ/mol, and 450.4 J/mol·K, respectively (Figure 1D). This complete thermodynamic information confirmed the interaction between FL-EK1 and HSA.

### 3.2. FL-EK1 Protein Potently Inhibited SARS-CoV-2 S Protein-Mediated Cell–Cell Fusion and Suppressed Both Pseudotyped and Authentic SARS-CoV-2 Infection

**Figure 2:**
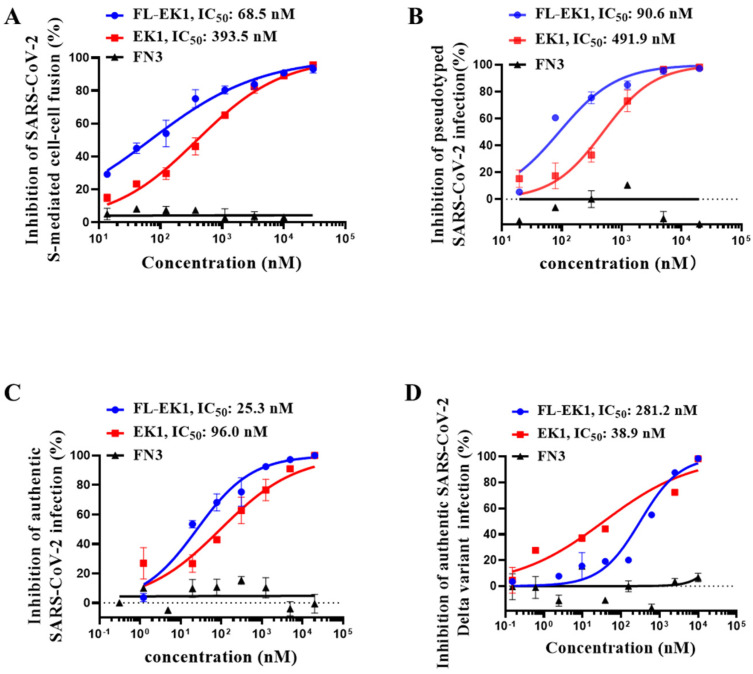
FL-EK1-mediated inhibition of SARS-CoV-2 infection. (**A**) FL-EK1-mediated inhibition of SARS-CoV-2 S-mediated cell–cell fusion. (**B**) FL-EK1-mediated inhibition of pseudotyped SARS-CoV-2. (**C**) FL-EK1-mediated inhibition of authentic SARS-CoV-2 infection. (**D**) FL-EK1-mediated inhibition of authentic SARS-CoV-2 Delta variant infection. Samples were tested in triplicate, and the experiment was repeated three times. Data from a representative experiment are presented as mean ± SD.

We then assessed the inhibitory activity of FL-EK1 (EK1 and FN3 as controls) on SARS-CoV-2 S protein-mediated cell–cell fusion. We found that FL-EK1 potently inhibited SARS-CoV-2 S protein-mediated cell–cell fusion in a dose-dependent manner with an IC_50_ (half maximal inhibitory concentration) of 68.5 nM, which is about 4.7-fold more potent than that of EK1 (IC_50_ = 393.5 nM), while FN3 showed no detectable inhibitory activity (Figure 2A). Then, we tested the inhibitory activity of FL-EK1 against SARS-CoV-2 PsV infection and found that FL-EK1 showed inhibitory activity with an IC_50_ value of 90.6 nM, which was about 4.4-fold more potent than that of EK1 (IC_50_ = 491.9 nM) (Figure 2B). We next evaluated the inhibitory activity of FL-EK1 against authentic SARS-CoV-2 infection. The results showed that FL-EK1 was also effective in inhibiting infection of authentic the SARS-CoV-2 wild-type (nCoV-SH01) strain with an IC_50_ value of 25.3 nM, about 2.8-fold over that of EK1 (IC_50_ = 96.0 nM) (Figure 2C). Furthermore, we assessed the inhibitory activity of FL-EK1 and EK1 against the authentic SARS-CoV-2 Delta variant with higher pathogenicity. We demonstrated that both FL-EK1 and EK1 could effectively inhibit infection of the authentic SARS-CoV-2 Delta variant in a dose-dependent manner with IC_50_ values of 281.2 and 38.9 nM, respectively (Figure 2D).

FN3 exhibited no inhibitory activity against S-mediated cell–cell fusion and none against pseudotyped and authentic SARS-CoV-2 infection (Figure 2A–D). The addition of FN3 to EK1 or FL-EK1 had no effect on the inhibitory activity of EK1 or FL-EK1 (data not shown).

Subsequently, we evaluated the inhibitory activity of FL-EK1 against infection by pseudotyped SARS-CoV-2 variants, including B.1.1.7 (Alpha), B.1.351 (Beta), P.1 (Gamma), B.1.617.2 (Delta), B.1.525 (Eta), B.1.617.1 (Kappa), C.37 (Lambda), and B.1.1.529 (Omicron). Our results showed that FL-EK1 and EK1 could also effectively inhibit infection by the pseudotyped SARS-CoV-2 variants with IC_50_ values ranging from 68.7 nM to 373 nM (see Table 1). 

The above results suggest that like EK1, FL-EK1 is also highly effective against infection of SARS-CoV-2 and its variants.

### 3.3. FL-EK1 Potently Inhibited S-Mediated Cell–Cell Fusion and Infection by Pseudotyped and Authentic HCoV-OC43 

**Figure 3:**
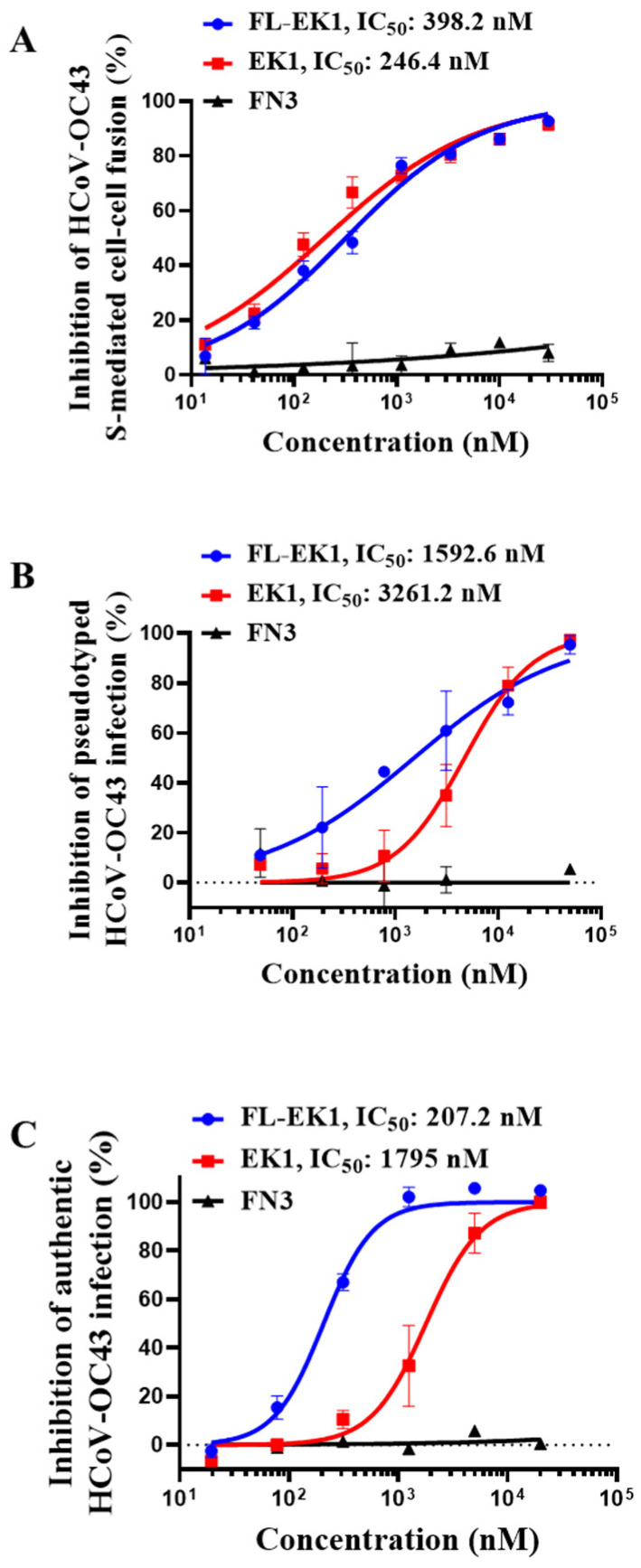
FL-EK1-mediated inhibition of HCoV-OC43 infection. (**A**) FL-EK1-mediated inhibition of HCoV-OC43 S-mediated cell–cell fusion. (**B**) FL-EK1-mediated inhibition of pseudotyped HCoV-OC43. (**C**) FL-EK1-mediated inhibition of authentic HCoV-OC43 infection. Samples were tested in triplicate, and the experiment was repeated three times. Data from a representative experiment are presented as mean ± SD.

Next, we evaluated the inhibitory activity of FL-EK1 and EK1 against cell–cell fusion mediated by the S protein of HCoV-OC43, which also belongs to β-CoV and can cause flu-like disease in HCoV-OC43-infected patients. We found that both FL-EK1 and EK1 were highly potent in inhibiting HCoV-OC43 S-mediated cell–cell fusion in a dose-dependent manner with an IC_50_ value of 398 and 246 nM, respectively (Figure 3A). We then evaluated the inhibitory activity of FL-EK1 and EK1 against HCoV-OC43 PsV infection. Similarly, both FL-EK1 and EK1 could effectively inhibit PsV infection with an IC_50_ value of 1593 and 3261 nM, respectively (Figure 3B). Finally, we assessed the antiviral activity of FL-EK1 and EK1 against authentic HCoV-OC43 infection. Again, both FL-EK1 and EK1 were effective in inhibiting authentic HCoV-OC43 infection in a dose-dependent manner with IC_50_ values of 207 and 1795 nM, respectively (Figure 3C). Like EK1, these results show that FL-EK1 also exhibits broad-spectrum antiviral activity against highly pathogenic SARS-CoV-2 and low-pathogenic HCoV-OC43.

### 3.4. FL-EK1 Exhibited Strong Protective Effects against Infection by Authentic HCoV-OC43 and SARS-CoV-2 Delta Variant in Mice

**Figure 4:**
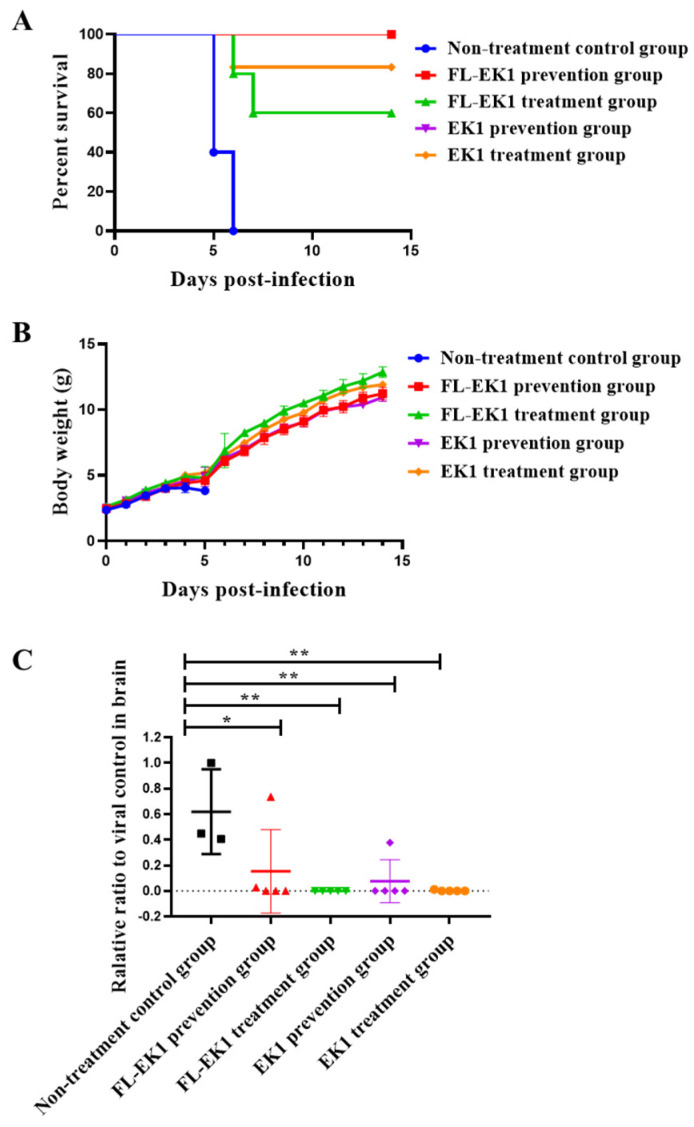
FL-EK1-mediated inhibition of authentic HCoV-OC43 infection in vivo. In vivo prophylactic and protective efficacy of FL-EK1 and EK1 against authentic HCoV-OC43 infection in ICR suckling mice via the intranasal route for FL-EK1 administration and viral challenge. Mouse survival rate (**A**) and bodyweight changes (**B**) were recorded. Viral titer in the brain was measured by real-time PCR (**C**). Samples were tested in triplicate, and the RT-PCR was repeated twice. Data from a representative experiment are presented as mean ± SD. * *p* < 0.05, ** *p* < 0.01.

Here, we investigated the in vivo prophylactic and therapeutic effects of FL-EK1 and EK1 on low-pathogenic authentic HCoV-OC43 infection in ICR suckling mice via the intranasal (IN) route for administration of FL-EK1 and viral challenge. We treated suckling mice with FL-EK1 and EK1 via the IN route at a dose of 10 mg/kg at 0.5 h before (for the prevention group) and 0.5 h after (for the treatment group) challenge with HCoV-OC43 at 100 TCID_50_ via the IN route. For the non-treatment control group, mice were not given a peptide, just PBS 0.5 h before viral challenge. After 14 days of observation, 100% of the mice in the non-treatment control group died, while 100% of the mice in both FL-EK1 and EK1 prevention groups were protected. The survival rates of the suckling mice in the EK1- and FL-EK1-treated groups were 83% and 60%, respectively (Figure 4A). The bodyweight of mice in the FL-EK1 and EK1 prevention and treatment groups increased slowly between day 4 and day 5 after infection but quickly returned to normal after day 6, while that of mice in the non-treatment group decreased rapidly on the fourth day after infection, and all died after the fifth day (Figure 4B). The changes of viral load in brain tissue of suckling mice on the fifth day after infection were also detected. In terms of viral RNA content in brain tissue of suckling mice, a significant difference was noted between the prevention group and the treatment group and the control group, with *p*-values less than 0.05 and 0.01, respectively (Figure 4C).

**Figure 5:**
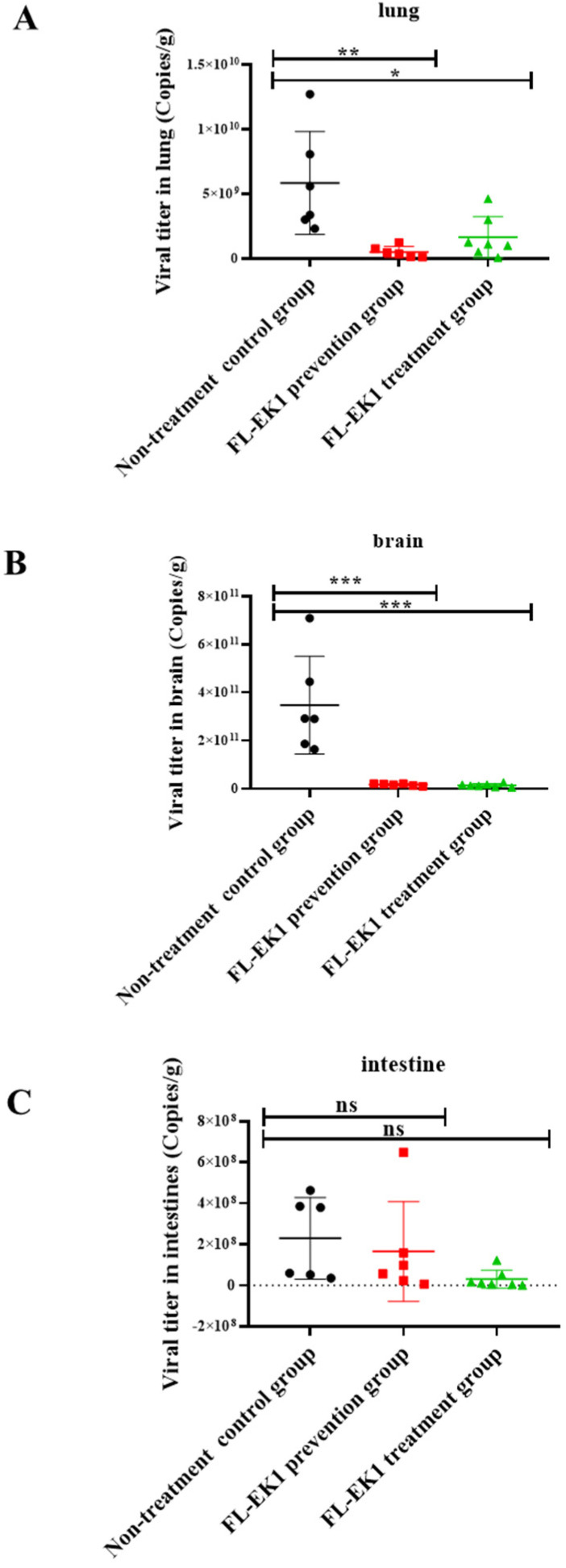
FL-EK1-mediated inhibition of authentic SARS-CoV-2 Delta variant infection in vivo. Prophylactic and protective efficacy of FL-EK1 against SARS-CoV-2 Delta variant infection in Tgtn mice via the intranasal route for FL-EK1 administration and viral challenge. Viral titer in lung (**A**), brain (**B**), and intestine (**C**) was measured by real-time PCR. Samples were tested in triplicate, and the RT-PCR was repeated twice. Data from a representative experiment are presented as mean ± SD. ns, no significance, * *p* < 0.05, ** *p* < 0.01, *** *p* < 0.001.

We further investigated the prophylactic and protective efficacy of FL-EK1 against authentic SARS-CoV-2 Delta variant infection in eight-week-old SPF Tgtn mice via the IN route for the administration of FL-EK1 and for viral challenge, as described above. The changes of viral yields in lung, brain, and intestinal tissues of mice on the fourth day post-infection were also examined. We found that viral yields in the lung of mice in the prevention and treatment groups were significantly lower than those in the non-treatment group with *p*-values less than 0.01 and 0.05, respectively (Figure 5A). Similarly, the viral yields in the brain of mice in the prevention and treatment groups were significantly lower than those in the non-treatment group with *p*-values less than 0.001 (Figure 5B). For viral yields in the intestinal tissues, no significant difference was noted between the prevention or treatment group and the non-treatment group (Figure 5C). All the above results indicate that FL-EK1 is effective in protecting mice against infection by the low-pathogenic HCoV-OC43 and the highly pathogenic SARS-CoV-2 Delta variant.

### 3.5. FL-EK1 Showed Improved Ex Vivo Antiviral Activity and Prolonged Serum Half-Life

**Figure 6:**
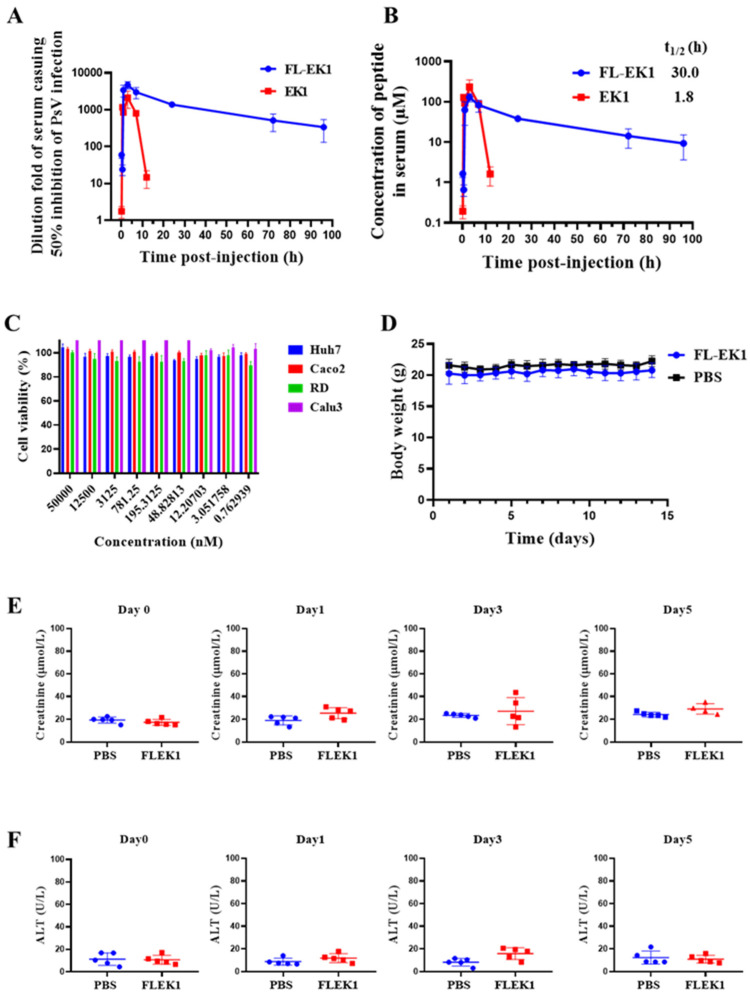
Ex vivo anti-SARS-CoV-2 PsV activity and concentrations of FL-EK1 and EK1 in mouse serum and evaluation of in vitro cytotoxicity and in vivo safety of FL-EK1. (**A**) Ex vivo anti-SARS-CoV-2 PsV activity of serum samples collected from mice at different time points after intraperitoneal injection of FL-EK1 and EK1 (*n* = 3). (**B**) Concentration of FL-EK1 and EK1 in mouse serum samples and the half-life of each were estimated. (**C**) In vitro cytotoxicity of FL-EK1 at the graded concentration on Huh7, Caco2, RD, and Calu3 cells. (**D**) Bodyweight changes of mice treated with FL-EK1 via the intranasal route. (**E**) Creatinine in serum samples of mice treated with FL-EK1 via intranasal administration was measured by using a creatinine assay kit. (**F**) ALT in serum samples of mice treated with FL-EK1 via intranasal administration was measured by an ALT assay kit. Samples were tested in triplicate, and the experiment was repeated three times. Data from a representative experiment are presented as mean ± SD.

Next, we evaluated whether FL-EK1 also enhances antiviral activity ex vivo, as determined by the highest serum dilution fold causing a 50% inhibition of viral infection (equivalent to IC_50_) induced by FL-EK1 and EK1 and by the extended in vivo half-life. Two groups of mice were injected intraperitoneally with 100 μL of FL-EK1 at 40mg/kg or EK1 at 8.25 mg/kg. Serum samples from mice at different time points were collected and stored separately before use. The inhibitory activity of FL-EK1 and EK1 against SARS-CoV-2 PsV infection was detected. As shown in Figure 6A, serum samples from mice injected with EK1 reached maximum inhibitory activity (about 2136-fold of their IC_50_) at 3 h post-injection and decreased to a very low level (about 14-fold of their IC_50_) at 12 h post-injection. However, serum samples of mice injected with FL-EK1 reached maximum inhibitory activity (about 4703-fold of their IC_50_) at 4.3 h post-injection and then slowly decreased to a relatively high level (about 338-fold of their IC_50_) at 96 h post-injection. 

Previous studies in the literature found a high correlation between the lipopeptide concentrations in sera of rats treated with lipopeptides and their in vitro anti-HIV-1 activity by Pearson’s Correlation Coefficient analysis [25]. In our previous study, the concentrations of CP24 and IBP-CP24 in rhesus monkey sera were measured by sandwich ELISA, and the half-life of each was calculated based on in vitro IC_50_ and ex vivo anti-HIV-1 activity. The results obtained by both methods were consistent [26]. Therefore, we estimated the concentrations of FL-EK1 and EK1 in sera collected at different time points based on the serum dilutions of FL-EK1 and EK1 for in vitro inhibition of SARS-CoV-2 PsV infection and the serum dilutions of FL-EK1 and EK1 for ex vitro anti-SARS-CoV-2 PsV infection according to previous experimental studies [27]. Next, we used MODFIT software [22] to calculate the half-life of FL-EK1 and EK1 based on serum concentration data and other pharmacokinetic parameters at different time points (Table 2). The serum half-life of FL-EK1 (t_1/2_ = 30 h) was about 15.7-fold longer than that of EK1 (t_1/2_ = 1.8 h). These results indicate that the half-life of EK1 conjugated with FN3 is significantly prolonged (Figure 6B).

### 3.6. FL-EK1 Exhibited No Significant In Vivo or In Vitro Toxicity

We then used the CCK8 assay to detect the potential in vitro cytotoxicity of FL-EK1 at gradient concentrations on Huh7, Caco2, RD, and Calu3 cells. As shown in Figure 6C, FL-EK1 showed no detectable in vitro cytotoxicity at concentrations up to 50 μM. The selectivity index (SI = CC_50_/IC_50_) of FL-EK1 was greater than 200, indicating that FL-EK1 is a long-acting HCoV fusion inhibitor with no cytotoxicity in vitro. 

We further evaluated the in vivo safety of FL-EK1 in mice. Mice in the FL-EK1 and PBS groups were given FL-EK1 and PBS via the intranasal route once daily for 3 days, respectively. Within two weeks after administration, the bodyweight of mice in both FL-EK1 and PBS groups was normal (Figure 6D). The mental state of the mice in both groups was also normal (data not shown). Serum creatinine and glutamic-pyruvate transaminase (ALT) levels in the sera of mice in each group were measured using the sera collected on day 1 before administration and days 1, 3, and 5 days after the last dose of FL-EK1, respectively. Levels of serum creatinine and ALT in the FL-EK1 group at all time points were similar to levels in the PBS control group (*p* > 0.05), indicating that FL-EK1 has a good safety profile in vivo (Figure 6E,F).

## 4. Discussion

The receptor of SARS-CoV and SARS-CoV-2 is angiotensin-converting enzyme 2 (ACE2) [28], which is mainly expressed in lung and small intestinal epithelial cells, but also expressed in tissues, such as the heart and kidney [29]. An HCoV particle expresses the S protein, a class I membrane fusion protein, consisting of S1 and S2 subunits [30,31,32]. The entry of HCoV into the host cell is initiated by binding the RBD in the S1 subunit to the ACE2 receptor on the cell surface, triggering the insertion of the fusion peptide (FP) into the cell membrane and exposure to the HR1 and HR2 in the S2 subunit. Then, the HR1 forms a trimer with which HR2 interacts to, in turn, form an anti-parallel six-helix bundle (6-HB), bringing viral and cell membrane together for fusion and entry of the viral genetic material into the cytoplasm of the host for replication [33,34,35,36]. Therefore, the HR1 trimer is an important target for the development of HCoV fusion/entry inhibitors.

Previously, we identified a pan-CoV fusion inhibitor, EK1, which can interact with the HR1 trimer in many HCoV spike proteins and block homologous viral 6-HB fusion core formation, thus demonstrating its efficacy in inhibiting fusion and infection of SARS-CoV, MERS-CoV, SARS-CoV-2, and other HCoVs [10]. Considering that its short half-life may limit its future clinical application, we originally planned to conjugate polyethylene glycol (PEG) to the N- or C-terminus of EK1 peptide in order to increase its half-life as we know that PEG modification of a peptide can reduce its spherical filtration, thereby increasing plasma half-life by prolonging elimination of the peptide [13]. However, we dismissed this plan since it was reported that the application of PEG in peptide- or protein-based injectable therapeutics could induce anti-PEG antibodies in vivo, thereby risking reduced efficacy of drugs or increasing adverse effects [37,38].

Other strategies involve binding to albumin in the circulation as a carrier to extend half-life and thus reduce the frequency of peptide therapy. The general principle of the above strategy is to reduce renal clearance by increasing molecular size (hydrodynamic volume) through post-translational modifications or adding components that extend half-life. For example, polymers or flexible polypeptide chains can be coupled to the target protein or certain fractions, such as the Fc region of IgG (IgG1, IgG2, and IgG4). Upon binding to albumin, an FcRn-mediated circulating process begins, thus further extending plasma half-life and improving bioavailability [12].

Given that the plasma protein HSA has a very long half-life (2–4 weeks), as well as large volume and recirculation of interactions with neonatal Fc receptor (FcRn) [39], recombinant therapeutic proteins may gain a longer half-life by reversibly binding to HSA through an albumin-binding motif or scaffold [15,40,41]. Because of its small size, exceptional thermal stability, solubility, good biophysical properties, abundance in human blood and extracellular matrix, and lack of toxicity or immunogenicity [15], FN3 has become one of the most widely used non-antibody albumin-binding scaffolds known to generate artificial binding proteins [16,17,18,23,42]. In addition, after the deletion of cysteine residues, the modified FN3 can be expressed at a high level in *E. coli* [15,24].

Therefore, in the present study, we designed and constructed the vector encoding the amino acid sequence of the engineered protein FL-EK1 consisting of the modified FN3 with reversible albumin-binding capacity, a 35-mer flexible linker (L35), and the pan-CoV fusion inhibitor EK1 (Figure 1B). FL-EK1 could be easily expressed in the *E. coli* system. The purified FL-EK1 retained its binding activity to HSA through its FN3 part and its capacity to interact with HR1P via its EK1 part to successfully block 6-HB formation between HR1P and HR2P (Figure 1). As with EK1 [10], FL-EK1 could effectively inhibit SARS-CoV-2 S-mediated cell–cell fusion and infection by both the pseudotyped and authentic SARS-CoV-2 and HCoV-OC43, as well as the SARS-CoV-2 Delta variant (Figure 2 and Figure 3). Similar to EK1 [10,20,43], FL-EK1 showed potent inhibitory activity against infection by pseudotyped SARS-CoV-2 variants, including Alpha, Beta, Gamma, Delta, Eta, Kappa, Lambda, and Omicron (see Table 1), confirming that the EK1 component of FL-EK1 maintains its broad-spectrum anti-HCoV activity. FL-EK1 could effectively protect mice from infection by HCoV-OC43 and SARS-CoV-2 Delta variant in vivo (Figure 4 and Figure 5), also consistent with the protective activity of EK1 [10,43]. Most importantly, FL-EK1 exhibited a substantially extended half-life (t_1/2_ = 30 h), compared with that of free EK1 peptide (t_1/2_ = 1.8 h) (Figure 6B), possibly because FL-EK1 could reversibly bind to HSA through its FN3 part in FL-EK1.

In conclusion, this novel albumin-binding, motif-conjugated pan-CoV fusion inhibitor, FL-EK1, can reversibly bind to HSA via its FN3 part and maintain its potent and broad-spectrum anti-HCoV activity via its EK1 part, resulting in remarkably extended half-life and improved pharmaceutical profile, thus overcoming the drawback of the regular peptide-based pan-CoV fusion inhibitor EK1 with its short half-life. In addition, FL-EK1, as a recombinant protein, can be produced in an *E. coli* system on a large scale, thus reducing production costs, compared with the EK1 peptide. Therefore, FL-EK1 is a promising candidate that can be further developed as a long-acting pan-CoV fusion inhibitor-based antiviral drug for prevention and treatment of infection by the current and future SARS-CoV-2 variants, as well as other emerging highly pathogenic HCoVs.

## Figures and Tables

**Table 1:** Inhibitory activity of FL-EK1 and EK1 against pseudotyped SARS-CoV-2 variants.

Pseudotyped SARS-CoV-2 Variants	IC_50_ (nM)	
	FL-EK1	EK1
B.1.1.7 (Alpha)	114.5 ± 33.4	69.0 ± 20.8
B.1.351 (Beta)	201.2 ± 16.8	109.9 ± 25.3
P.1 (Gamma)	373.1 ± 16.9	191.3 ± 17.0
B.1.617.2 (Delta)	133.0 ± 16.5	190.6 ± 29.0
B.1.525 (Eta)	179.2 ± 38.3	135.6 ± 11.2
B.1.617.1 (Kappa)	230.9 ± 49.3	107.1 ± 7.3
C.37 (Lambda)	88.5 ± 58.2	68.7 ± 25.4
B.1.1.529 (Omicron)	297.5 ± 188.2	236.8 ± 10.6

Samples were tested in triplicate, and the experiment was repeated three times. Data from a representative experiment are presented as mean ± SD.

**Table 2:** Pharmacokinetic parameters of FL-EK1 and EK1 by intraperitoneal injection in mice.

Peptide	T_1/2_ (h)	Tmax (h)	Cmax (μg/mL)	Lin/Log AUC (μg/mL/h)
FL-EK1	30.0 ± 12.8	4.3 ± 1.9	2918.5 ± 864.5	57,309.2 ± 9831.1
EK1	1.8 ± 1.0	3.0 ± 0.0	1011.8 ± 704.4	4628.9 ± 1684.9

Samples were tested in triplicate, and the experiment was repeated three times. Data from a representative experiment are presented as mean ± SD.

## Data Availability

The raw data of this paper are available on request from the corresponding author.
